# Comparison of the 2023 ISCVID and ESC Duke clinical criteria for the diagnosis of infective endocarditis among patients with positive blood cultures for new typical microorganisms

**DOI:** 10.1007/s15010-024-02460-1

**Published:** 2025-01-02

**Authors:** Nicolas Fourré, Virgile Zimmermann, Laurence Senn, Pierre Monney, Georgios Tzimas, Florian Tagini, Piergiorgio Tozzi, Matthias Kirsch, Benoit Guery, Matthaios Papadimitriou-Olivgeris

**Affiliations:** 1 Infectious Diseases Service, Lausanne University Hospital, Lausanne, Switzerland; 2 Infection Prevention and Control Unit, Lausanne University Hospital, Lausanne, Switzerland; 3 Department of Cardiology, Lausanne University Hospital and University of Lausanne, Lausanne, Switzerland; 4 Institute of Microbiology, Lausanne University Hospital, Lausanne, Switzerland; 5 Department of Cardiac Surgery, Lausanne University Hospital and University of Lausanne, Lausanne, Switzerland; 6 Infectious Diseases Service, Cantonal Hospital of Sion and Institut Central des Hôpitaux (ICH), Sion, 1951 Switzerland

**Keywords:** Candidaemia, Duke criteria, Infective endocarditis, Bloodstream infection, *Pseudomonas aeruginosa*

## Abstract

**Purpose:**

To evaluate the performance of the Duke clinical criteria of the European Society of Cardiology (ESC; 2015 and 2023 versions) and the 2023 International Society for Cardiovascular Infectious Diseases (ISCVID) in diagnosing infective endocarditis (IE) among patients with bacteraemia/candidaemia by pathogens introduced for the first time as typical microorganisms by ISCVID.

**Methods:**

Retrospective study.

**Setting:**

This study included adult patients with bacteraemia/candidaemia by such pathogens (coagulase negative staphylococci, *Abiotrophia* spp., *Gemella* spp., and *Granulicatella* spp., *Cutibacterium. acnes*, *Corynebacterium striatum*, *C. jeikeium*, *Pseudomonas aeruginosa*, *Serratia marcescens*, non-tuberculous mycobacteria, and *Candida* spp.) hospitalized at Lausanne University Hospital. Episodes were classified as IE by two expert clinicians.

**Results:**

Among 463 episodes with bacteraemia/candidaemia by such pathogens, IE was diagnosed in 63 episodes (14%). IE prevalence was 17% among episodes with bacteraemia by *Staphylococcus lugdunensis* or *Abiotrophia spp*. No case of IE was identified among *Granulicatella* spp. and *Gemella* spp. bacteraemias. Among 113 episodes with intracardiac prosthetic material, IE prevalence was 51% in episodes with bacteraemia by *S. epidermidis*. Sensitivity for the 2015 Duke-ESC, 2023 Duke-ISCVID, and the 2023 Duke-ESC clinical criteria was calculated at 5%, 57%, and 8%, respectively. More episodes were classified as possible IE by the 2023 Duke-ISCVID (30%) compared to 2015 Duke-ESC (13%) and 2023 Duke-ESC (16%) clinical criteria.

**Conclusion:**

The 2023 ISCVID version demonstrated superior sensitivity compared to both 2015 and 2023 Duke-ESC in diagnosing IE caused by new typical microorganisms, compared to the other criteria, albeit an increase in cases being classified as possible IE.

**Supplementary Information:**

The online version contains supplementary material available at 10.1007/s15010-024-02460-1.

## Introduction

From the inception of the Duke criteria, the major microbiological criterion consisted of multiple positive blood cultures from typical pathogens commonly associated with infective endocarditis (IE), including *Staphylococcus aureus*, certain streptococci, and enterococci [1–3].

In 2023, the International Society for Cardiovascular Infectious Diseases (ISCVID) revisited the criteria. Additional bacteria were classified as typical microorganisms based on research findings spanning over the last two decades [4]. Examples include *S. lugdunensis*, an infrequent cause of bacteraemia that exhibits virulence similarities to *S. aureus*, and streptococci-like bacteria such as *Abiotrophia* spp., *Granulicatella* spp., and *Gemella* spp., which are even rarer causes of bacteraemia but have been linked to a relatively high risk of IE [5–7]. Indeed, *Abiotrophia* spp., *Granulicatella* spp. were considered as typical IE microorganisms in the initial Duke criteria [2], as they were classified at that time as nutritionally variant streptococci. However, subsequent research revealed significant phylogenetic distances between these species and other members of the genus *Streptococcus* [8].

Moreover, certain microorganisms infrequently associated with IE were discovered to be more prevalent in patients with prosthetic intracardiac material, such as prosthetic valves, including transcatheter aortic valve implantation (TAVI) and cardiac implantable electronic devices (CIED) [4, 9–12]. Consequently, a new category of typical microorganisms was established, applicable only in the presence of such intracardiac prosthetic material. This category encompassed coagulase-negative staphylococci (CoNS), *Cutibacterium acn*es, *Corynebacterium striatum* and *C. jeikeium*, *Pseudomonas aeruginosa*, *Serratia marcescens*, non-tuberculous mycobacteria, and *Candida* spp.

Later in the same year, the European Society of Cardiology (ESC) published an additional version of the Duke criteria that did not include the aforementioned pathogens as typical microorganisms [13]. The 2023 iterations of the Duke criteria led to an increase in sensitivity for diagnosing IE compared to the 2015 version, regardless of the infecting pathogen [14–20].

Our study aims to assess the performance of the different versions of the Duke clinical criteria (2015 Duke-ESC, 2023 Duke-ESC, and 2023 Duke-ISCVID) for diagnosing IE in patients with bacteraemia/candidaemia by new typical microorganisms (CoNS, *Abiotrophia* spp., *Gemella* spp., and *Granulicatella* spp., *C. acn*es, *C. striatum*, *C. jeikeium*, *P. aeruginosa*, *S. marcescens*, non-tuberculous mycobacteria, and *Candida* spp.) proposed in the 2023 Duke-ISCVID [4].

## Materials and methods

This retrospective study was conducted at Lausanne University Hospital in Switzerland from January 2014 to March 2023. The study integrated data from two separate cohorts: bacteraemia/candidaemia cohort (January 2015 to December 2021), and non-duplicate episodes from the cohort of patients with suspected IE (January 2014 to June 2023; suspicion of IE was defined as blood cultures drawn and echocardiography performed specifically for the research of IE).

Included microorganisms were divided in two Groups. Group A comprised of microorganisms that were considered typical regardless of the presence of intracardiac material (*S. lugdunensis*, *Granulicatella* spp., *Abiotrophia* spp., *Gemella* spp.), and Group B consisted of those that we considered typical only in the presence of intracardiac material (CoNS other than *S. lugdunensis*, *C. acnes*, *C. striatum*, *C. jeikeium*, *P. aeruginosa*, *S. marcescens*, non-tuberculous mycobacteria, and *Candida* spp.).

Inclusion criteria encompassed adult patients (≥ 18 years old) with at least one positive blood culture for.

- any Group A microorganism, or.
- any Group B microorganism along with either presence of intracardiac prosthetic material or cardiac imaging studies performed.

Exclusion criteria included patients who had formally declined the use of their data, cases with incomplete medical records (including patients transferred to other hospitals at the onset of infection without follow-up data), and instances where isolated microorganisms were considered contaminants (if only one positive blood culture set grew a skin contaminant).

Data on demographics (age, sex), demographic, clinical, imaging, microbiological, surgical, and pathological were retrieved from patient’s electronic health charts.

Blood cultures bottles (aerobic: BD Bactec Plus aerobic/F; anaerobic: BD Bactec Lytic/10 anaerobic/F) were drawn and incubated in a BACTEC FX 400^®^ (BD, Becton Dickinson, Franklin Lakes, NJ, US) for 14 days when endocarditis or candidaemia were suspected (if not labeled, the blood cultures were incubated for 5 days). Species identification in the species level was performed directly from blood culture pellets as well as from subcultures using matrix-assisted laser desorption-ionization time of flight mass spectrometry (Bruker Daltonics Inc., Billerica, MA, USA). From 2014 to 2018, blood culture pellets were obtained using the method described by Croxatto *et a*l. and since 2019, the rapidBAC PROTM II (Nittobo, Tokyo, Japon) was used [21, 22]. When MALDI-TOF score was inconclusive (< 1.8 for blood culture pellets or < 2 for subculture colonies), a protein extraction was performed before analysing the spectra MALDI-TOF again. Briefly, 25 µl of blood culture pellet or ~ 1 µl of colonies were resuspended in 500 µl of sterile water at 13,000 g for 2 min at room temperature. After discarding the supernatant, the pellet was resuspended with 70% formic acid, followed by the addition of 100% acetonitrile and vortexing for one minute. After another centrifugation under the same conditions, the sample was prepared for analysis by drying two deposits on a target plate at 35 °C and adding 1 µl of matrix before the final drying step.

When there was a suspicion of mycobacterial infection, lithium heparinate samples were drawn from patients and subsequently incubated in BD Bactec Myco/F-Lytic culture vials for 42 days (BD, Becton Dickinson, Franklin Lakes, NJ, US). Mycobacteria species identification was then performed using a 16 S rRNA PCR and Sanger sequencing as previously described [23].

In our institution, notifications regarding patients with positive blood cultures were received by infectious diseases (ID) consultants, but the decision to conduct an ID consultation was left to their discretion. However, for all patients with candidaemia or those with suspected IE, an ID consultation was mandatory. In January 2018, an Endocarditis Team was established, and since then, the diagnosis of IE has been determined by the Endocarditis Team. For all cases prior to 2018, the IE diagnosis (reference standard) was made by two IE experts (MPO and PM) in an *a posteriori* and consensus approach, after reviewing clinical, laboratory, microbiological, imaging, surgical, and histopathological findings from patients’ medical records, and not on the classification of the Duke criteria. To ensure consistency and continuity in diagnosis, both expert clinicians were members of the Endocarditis Team. Cases were further categorized as rejected, possible, or definite IE according to the three versions of the Duke clinical criteria (2015 Duke-ESC [1], 2023 Duke-ESC [13], and 2023 Duke-ISCVID [4]).

The date of collection of the first positive blood culture was defined as the onset of infection. A new episode was included if more than 60 days had passed from the first negative blood culture for the initial bacteraemia/candidaemia. True bacteremia was defined as the identification of a common skin contaminant isolated from at least two separate blood culture sets, combined with the decision by the patient’s physician to treat the identified pathogen. The determination of the infection site was based on the assessment by the ID consultant, taking into account clinical, radiological, microbiological, and operative findings.

SPSS version 26.0 (SPSS, Chicago, IL, USA) was used for data analyses. Categorical variables were analyzed using the *chi*-square or Fisher exact test and continuous variables with Mann–Whitney *U* test. The efficacy of each version of the Duke clinical criteria was evaluated by measuring the level of agreement between the reference standard and either definite IE cases or definite/possible IE cases. Sensitivity, specificity, positive and negative predictive values (PPV, NPV) and accuracy were calculated with a 95% confidence interval (CI). All tests were 2-tailed, and a significance level of *P* < 0.05 was applied.

## Results

**Fig. 1:**
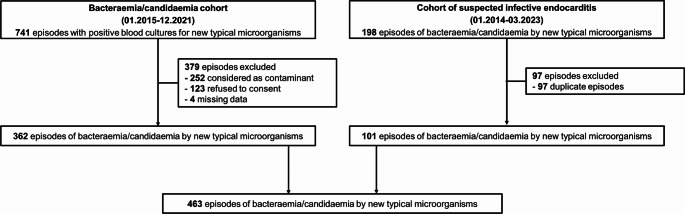
Flowchart of included episodes

Among the 939 episodes with positive blood cultures for new typical pathogens, 463 episodes were included; 362 from the bacteraemia/candidaemia cohort, and 101 non-duplicate episodes from the cohort of suspected IE (Fig. 1). The most frequently isolated microorganism was *Candida* spp. (128; 28%), followed by *S. epidermidis* (125; 27%) and *P. aeruginosa* (94; 20%) (Table 1).

**Table 1:** Comparison of episodes with or without infective endocarditis among 462 episodes with positive blood cultures for new typical microorganisms

	Bacteremia/candidaemia without evidence of infective endocarditis (*n* = 400)	Infective endocarditis (*n* = 63)	*P*
Demographics			
Male sex	278 (70%)	43 (68%)	0.883
Age (years)	65 (55–75)	71 (61–80)	0.020
Cardiac predisposing factors			
Intravenous drug use	17 (4%)	1 (2%)	0.489
Rheumatic heart disease/Hypertrophic cardiomyopathy	1 (0.3%)	1 (2%)	0.254
Congenital disease	3 (0.8%)	7 (11%)	< 0.001
Prosthetic valve	46 (12%)	19 (30%)	< 0.001
Prior endocarditis	11 (3%)	10 (16%)	< 0.001
Moderate/severe valve regurgitation/stenosis	38 (10%)	19 (30%)	< 0.001
CIED	37 ()9%	19 (30%)	< 0.001
Transcatheter aortic valve replacement	6 (2%)	3 (5%)	0.110
Heart transplantation	9 (2%)	1 (2%)	1.000
Left ventricular assist device	5 (1%)	0 (0%)	1.000
Microbiological data			
Two or more blood cultures positive (initial blood cultures)	251 (63%)	56 (89%)	< 0.001
Three or more blood cultures positive (initial blood cultures)	62 (16%)	16 (25%)	0.069
*S. lugdunensis*	21 (5%)	7 (11%)	0.085
*Granulicatella spp.*	25 (6%)	0 (0%)	0.035
*Abiotrophia spp.*	6 (2%)	4 ()6%	0.035
*Gemella spp.*	15 (4%)	0 (0%)	0.241
*S. epidermidis*	92 (23%)	33 (52%)	< 0.001
Coagulase negative staphylococci other than *S. lugdunensis* and *S. epidermidis*	54 (14%)	4 ()6%	0.150
*Cutibacterium acnes*	2 (0.5%)	2 (3%)	0.092
*P. aeruginosa*	90 (23%)	4 (6%)	0.002
*S. marcescens*	29 ()7%	0 (0%)	0.022
Non-tuberculous mycobacteria	3 (0.8%)	0 (0%)	1.000
*Candida spp.*	119 (30%)	9 (14%)	0.010
Polymicrobial bloodstream infection	83 (21%)	2 (3%)	< 0.001
Other typical pathogens	31 (8%)	1 (2%)	0.104
Infection data			
Fever	337 (84%)	47 (75%)	0.071
Sepsis	190 (48%)	24 (38%)	0.176
Vascular phenomena (major arterial emboli, septic pulmonary infarcts, mycotic aneurysm, intracranial hemorrhage, conjunctival hemorrhages, and Janeway’s lesions)	28 (7%)	14 (22%)	< 0.001
Immunological phenomena	7 (0.8%)	1 (2%)	0.430
Septic arthritis	2 (0.5%)	0 (0%)	1.000
Spondylodiscitis	7 (2%)	2 (3%)	0.352
Imaging data			
Positive echocardiography (either TTE or TEE) forvegetation, perforation, dehiscence of prothesis, abscess, aneurysm, pseudoaneurysm, fistula	0 (0%)	38 (60%)	< 0.001
Abnormal metabolic activity in ^18^F-FDG PET/CT	0 (0%)	13 (21%)	< 0.001
Abnormal metabolic activity in ^18^F-FDG PET/CT in native valve or CIED lead	0 (0%)	7 (11%)	< 0.001
Abnormal metabolic activity in ^18^F-FDG PET/CT in prosthetic valve	0 (0%)	6 (10%)	< 0.001
Positive cardiac-CT for vegetation, perforation, dehiscence of prothesis, abscess, aneurysm, pseudoaneurysm, fistula	0 ()0%	3 (5%)	< 0.001
Leaflet thickening in echocardiography or cardiac-CT	6 (2%)	12 (19%)	< 0.001
Significant new valvular regurgitation	1 (0.3%)	10 (16%)	< 0.001
Data on surgery/CIED-extraction/histopathology			
Valve surgery performed	0 ()0%	22 (35%)	< 0.001
Macroscopic evidence of IE by inspection	0 (0%)	21 (33%)	< 0.001
CIED-extraction (among 59 episodes with CIED)	1 (0.3%)	12 (63%)	< 0.001
Autopsy performed	1 (0.3%)	0 (0%)	1.000
Duke pathological criterion	0 (0%)	21 (33%)	< 0.001

Data are depicted as number (percentage) or median (Q1-3)

^18^F-FDG PET/CT: ^18^F-Fluorodeoxyglucose Positron Emission Tomography/Computed Tomography; CIED: cardiac implantable electronic device; TEE: transesophageal echocardiography; TTE: transthoracic echocardiography

Sixty-three (14%) episodes were diagnosed with IE (reference standard), with 34 (54%) involving native valves, 23 (37%) prosthetic valves, 12 (19%) CIED-lead, and 1 (2%) other intracardiac structure. Diagnosis of IE was made at a median of three days (interquartile range: 2–6 days) from the first positive blood culture. Other foci of bacteraemia/candidaemia included catheter-related infection (96; 27%), bacteraemia/candidaemia of unknown origin (98; 27%), abdominal (44; 12%), low-respiratory tract (38; 11%), urinary-tract (22; 6%), bone and joint (16; 4%), skin and soft tissue (11; 3%), and other types of infection (42; 12%).

Transthoracic, transesophageal echocardiography (TTE, TEE), ^18^F-FDG PET/CT, and cardiac CT were performed in 374 (81%), 107 (23%), 50 (11%), and 7 (2%) episodes, respectively, with 405 (88%) episodes undergoing at least one type of cardiac imaging. Among the 58 (13%) episodes without any cardiac imaging study, 55 (95%) were treated for less than 10 days of antimicrobial treatment.

The prevalence of the IE based on different microorganisms of Group A was evaluated in the 362 episodes from the bacteraemia/candidaemia cohort (Supplementary Table 1). IE prevalence was high among *S. lugdunensis* (4 out of 23 episodes; 17%), and *Abiotrophia spp.* (1 out of 6; 17%). No case of IE was identified among 21 and 14 episodes bacteraemia by *Granulicatella* spp. and *Gemella* spp., respectively. The prevalence of the IE based on different microorganisms of Group B was evaluated in the 113 episodes with intracardiac prosthetic material (Supplementary Table 2). IE prevalence was high in episodes with bacteraemia by *C. acnes* (2 out of 3; 67%), by *S. epidermidis* (19 out of 37; 51%), CoNS other than *S. lugdunensis* and *S. epidermidis* (1 out of 7; 14%), and candidaemia (7 out of 27; 26%). Six episodes (1%) presented a recurrence of IE by the same pathogen as the initial episode (2. *C. albicans*, 1 *S. epidermidis*, 1 *S. lugdunensis*, 1 *C. glabrata*, 1 *P. aeruginosa*).

Out of 463 episodes with bacteraemia/candidaemia by new typical microorganisms, 4 (0.9%), 41 (9%), and 7 (2%) episodes were classified as definite IE by the 2015 Duke-ESC, 2023 Duke-ISCVID, and 2023 Duke-ESC clinical criteria, respectively (Table 2). Using the 2023 Duke-ISCVID, more episodes with IE achieved the major microbiological criterion (40; 64%) compared to both ESC criteria (1; 2%). This led to an increase of episodes being classified as possible IE by the 2023 Duke-ISCVID (30%) compared to 2015 Duke-ESC (13%) and 2023 Duke-ESC (16%) clinical criteria. Table 3 provides an overview of the performance of the different versions of the Duke clinical criteria by measuring the level of agreement between the reference standard and definite IE cases. Sensitivity for the 2015 Duke-ESC, 2023 Duke-ISCVID, and the 2023 Duke-ESC clinical criteria was calculated at 5% (95% CI: 1–13%), 57% (44–70%), and 8% (3–18%), respectively, with specificity at 100% (99–100%), 99% (97–100%), and 100% (98–100%), respectively. Supplementary Table 3 shows the performance of the different versions of the Duke clinical criteria. by measuring the level of agreement between the reference standard and definite/possible IE cases.

**Table 2:** Classifications based on the three versions of the Duke clinical criteria

	Bacteremia/candidaemia without evidence of infective endocarditis (*n* = 400)	Infective endocarditis (*n* = 63)	*P*
Duke major clinical criteria			
Major imaging criterion (2015 ESC)	0 (0%)	46 (73%)	< 0.001
Major imaging criterion (2023 ISCVID)	1 (0.1%)	47 (75%)	< 0.001
Major imaging criterion (2023 ESC)	6 (2%)	52 (83%)	< 0.001
Major surgery criterion (2023 ISCVID)	0 (0%)	0 (0%)	-
Major microbiological criterion (2015 ESC)	17 (4%)	1 (2%)	0.489
Major microbiological criterion (2023 ISCVID)	85 (21%)	40 (64%)	< 0.001
Major microbiological criterion (2023 ESC)	17 (4%)	1 (2%)	0.489
Duke minor clinical criteria			
Minor microbiological criterion (2015 ESC)	14 (4%)	0 (0%)	0.234
Minor microbiological criterion (2023 ISCVID)	105 (26%)	7 (11%)	0.007
Minor microbiological criterion (2023 ESC)	14 (4%)	0 (0%)	0.234
Minor predisposition criterion (2015 ESC)	59 (15%)	31 (49%)	< 0.001
Minor predisposition criterion (2023 ISCVID)	116 (29%)	53 (84%)	< 0.001
Minor predisposition criterion (2023 ESC)	99 (25%)	43 (68%)	< 0.001
Minor vascular criterion (2015 ESC)	28 (7%)	14 (22%)	< 0.001
Minor vascular criterion (2023 ISCVID)	28 (7%)	14 (22%)	< 0.001
Minor vascular criterion (2023 ESC)	34 (9%)	15 (24%)	0.001
Minor immunological criterion (all versions)	6 (2%)	1 (2%)	1.000
Minor fever criterion (all versions)	337 (84%)	47 (75%)	0.071
Classification according to 2015 Duke-ESC clinical criteria			
Rejected	377 (94%)	21 (33%)	
Possible	22 (6%)	39 (62%)	
Definite	1 (0.3%)	3 (5%)	< 0.001
Classification according to 2023 Duke-ISCVID clinical criteria			
Rejected	277 (69%)	5 (8%)	
Possible	118 (30%)	22 (35%)	
Definite	5 (1%)	36 (57%)	< 0.001
Classification according to 2023 Duke-ESC clinical criteria			
Rejected	368 (92%)	13 (21%)	
Possible	30 (8%)	45 (71%)	
Definite	2 (0.5%)	5 (8%)	< 0.001

Data are depicted as number (percentage) or median (Q1-3)

ESC: European Society of Cardiology; ISCVID: International Society of Cardiovascular Infectious Diseases

**Table 3:** Diagnostic performance of the three versions of the Duke clinical criteria for the diagnosis of IE by measuring the level of agreement between the reference standard and definite IE cases

	Sensitivity % (95% CI)	Specificity % (95% CI)	PPV % (95% CI)	NPV % (95% CI)	Accuracy % (95% CI)
2015 Duke-ESC	5 (1–13)	100 (99–100)	75 (24–97)	87 (86–88)	87 (83–90)
2023 Duke-ISCVID	57 (44–70)	99 (97–100)	88 (75–95)	94 (92–95)	93 (90–95)
2023 Duke-ESC	8 (3–18)	100 (98–100)	71 (33–93)	87 (86–88)	87 (84–90)

ESC: European Society of Cardiology; IE: infective endocarditis; ISCVID: International Society of Cardiovascular Infectious Disease

## Discussion

The 2023 Duke-ISCVID clinical criteria demonstrated higher sensitivity compared to both ESC versions of the Duke clinical criteria, and this improvement can be primarily attributed to the changes made to the typical microorganisms by ISCVID. The results contrast those from previous evaluations of the 2023 versions of Duke criteria that showed comparable improvement of sensitivity [14, 17]; this discrepancy can be explained that these studies were conducted among patients with *S. aureus* bacteraemia [14] or those with suspected IE [17], whereas pathogens evaluated in the present study represented a small percentage of IE cases.

Furthermore, as previously shown [14–18, 20, 24], while sensitivity improved with the 2023 Duke-ISCVID, that was at expense of overall cases being classified as possible IE, thus if they would be applied in clinical practice, more cases would warrant clinical evaluation for them to diagnose IE or reject the diagnosis.

Despite the aforementioned improvement in sensitivity, not all included species exhibited the same risk of IE. Among the species included as typical microorganisms, irrespective of the presence of intracardiac prosthetic material, *S. lugdunensis* and *Abiotrophia* spp. showed a high risk of IE. Although classified among the CoNS, *S. lugdunensis* was found to be associated with a risk for IE development comparable to that of *S. aureus* [6, 7].

Conversely, among the cases of bacteraemia caused by *Granulicatella* spp. and *Gemella* spp. in the bacteraemia/candidaemia cohort, no patient had IE. In a previous study involving *Streptococcus*-like bacteria, *Abiotrophia* spp. exhibited the highest rate of IE among bacteraemia cases (4 out of 19; 21%), compared to *Gemella* spp. (6 out of 87; 7%) and *Granulicatella* spp. (9 out of 124; 7%) [5]. The absence of IE cases in our cohort could be explained by the low number of bacteraemias from such pathogens.

The microbiologic etiology of early prosthetic valve IE (within one year from valve implantation), including TAVI, differs from that of native valve IE, with higher rates of CoNS, Gram-negative bacilli, and *Candida* spp [10–12, 25, 26]. These differences in the microbiological profile of early prosthetic valve IE led to the addition of the typical microorganisms proposed by ISCVID in the presence of prosthetic intracardiac material. In previous studies on patients with prosthetic valve IE, CoNS were responsible for 10 to 27% of cases [10–12]. In the present study, approximately half of the bacteraemias caused by CoNS in patients with prosthetic intracardiac material had IE. Therefore, in patients with intracardiac prosthetic material and true bacteraemia caused by such bacteria, there should be a formal assessment to rule out infection of the prosthetic material.

Another notable observation was the elevated prevalence of IE attributed to *Candida* spp. in patients with candidaemia in the presence of prosthetic intracardiac material, as almost one-fourth of patients with candidaemia developed such a complication. Although IE is infrequent in patients lacking intracardiac prosthetic material, the presence of such material in patients with candidaemia should trigger cardiac imaging studies for IE investigation [10, 11]. IE caused by *Candida* spp. is linked to increased mortality, and valve surgery is deemed necessary, making early recognition crucial [13].

Other microorganisms included by ISCVID as typical microorganisms in the presence of intracardiac prosthetic material were rarely found to cause IE in such patients. Particularly, the inclusion of *P. aeruginosa* and *S. marcescens* in this category was based on a publication reporting that the risk of CIED-related infection in patients with bacteraemia caused by these organisms was comparable to that of *S. aureus* [27]. However, that study included all types of CIED-related infections, including pocket infections. Notably, while 50% of patients with bacteraemia due to *P. aeruginosa* or *S. marcescens* had a CIED-related infection, only 21% had positive echocardiographic findings suggestive of CIED-lead IE. However, subsequent studies, including ours, did not replicate this finding, revealing a low risk of IE for prosthetic intracardiac material, be it prosthetic valve or CIED, among patients with bacteraemia caused by aforementioned bacteria [10–12, 28, 29]. Studies focusing on prosthetic valve IE or CIED-lead IE caused by Gram-negative bacilli did not identify *P. aeruginosa* or *S. marcescens* as the most frequent culprits [10, 28, 29].

Our study was not without limitations. First, it was a retrospective single-center study and was not sufficiently large to assess the impact on diagnostic performance of the different versions of Duke criteria of less frequent microorganisms. However, to the best of our knowledge, this is the only study to focus on patients with positive blood cultures for new typical microorganisms, as defined by ISCVID. The inclusion of episodes from the cohort of patients with suspected IE could have led to an overrepresentation of cases at high risk for IE. To address this bias, we conducted an analysis of the subgroup of episodes from the bacteraemia cohort. However, in 58 (13%) episodes no cardiac imaging study was performed. The majority of these (55 episodes) had received less than 10 days of antibiotic treatment, which was considered insufficient to treat IE. Another constraint arises due to the lack of a universally accepted gold standard for diagnosing IE. In this study, we employed evaluation by two experienced physicians in IE as a reference standard, trying to mitigate this bias. Furthermore, we did not include *Streptococcus agalactiae* and *S. dysgalactiae* in the present study, since it would be more appropriate if they were evaluated alongside other streptococci [20]. 

In conclusion, the incorporation of new pathogens as typical microorganisms by the 2023 Duke-ISCVID resulted in increased sensitivity compared to both the 2015 and 2023 Duke-ESC, primarly attributed to the aforementioned additions. Although, this came at the cost of 30% of episodes being classified as possible IE by 2023 Duke-ISCVID. However, not all additions had the same impact, with the inclusion of *S. lugdunensis* irrespectively of the presence of intracardiac material, and CoNS and *Candida* spp. in the presence of intracardiac material having the most significant impact. Future evaluations of the ISCVID proposed changes in different settings could elucidate the impact of proposed changes before widespread implementation in clinical practice.

## Electronic supplementary material

Below is the link to the electronic supplementary material.

Supplementary Material 1

## Data Availability

The data that support the findings of this study are available from the corresponding author upon reasonable request.
