# Supplementary material for: Comparison of the 2023 ISCVID and ESC Duke clinical criteria for the diagnosis of infective endocarditis among patients with positive blood cultures for new typical microorganisms
Source: Infection. 2025 Jan 2;53(4):1393–401. doi: 10.1007/s15010-024-02460-1 (PMC12316761; doi:10.1007/s15010-024-02460-1)
Supplement: Supplementary file 1 — Supplementary Material 1 [file 15010_2024_2460_MOESM1_ESM.docx]

**Supplementary Table 1.** Prevalence of infective endocarditis of different microorganisms among 362 episodes from the bacteremia/candidemia cohort

|  | **Bacteremia/candidaemia without evidence of infective endocarditis (n=332)** | **Infective endocarditis (n=30)** | **Prevalence of infective endocarditis** |
| --- | --- | --- | --- |
| Group A microorganisms |  |  |  |
| *S. lugdunensis* | 19 (6%) | 4 (13%) | 17% |
| *Granulicatella* spp. | 21 (6%) | 0 (0%) | 0% |
| *Abiotrophia* spp. | 5 (2%) | 1 (3%) | 17% |
| *Gemella* spp. | 14 (4%) | 0 (0%) | 0% |
| Group B microorganisms |  |  |  |
| *S. epidermidis* | 63 (19%) | 17 (58%) | 21% |
| Coagulase negative staphylococci other than *S. lugdunensis* and *S. epidermidis* | 47 (14%) | 3 (10%) | 6% |
| *C. acnes* | 2 (0.6%) | 0 (0%) | 0% |
| *P. aeruginosa* | 76 (23%) | 1 (3%) | 1% |
| *S. marcescens* | 28 (8%) | 0 (0%) | 0% |
| Non-tuberculous mycobacteria | 3 (0.9%) | 0 (0%) | 0% |
| *Candida* spp. | 108 ()33% | 4 (13%) | 4% |

Data are depicted as number (percentage)

**Supplementary Table 2.** Prevalence of infective endocarditis of different pathogens among 113 episodes with intracardiac prosthetic material

|  | **Bacteremia/candidaemia without evidence of infective endocarditis (n=79)** | **Infective endocarditis (n=34)** | **Prevalence of Infective endocarditis** |
| --- | --- | --- | --- |
| Group A microorganisms |  |  |  |
| *S. lugdunensis* | 0 (0%) | 3 (9%) | 100% |
| *Granulicatella* spp. | 3 (4%) | 0 (0%) | 0% |
| *Abiotrophia* spp. | 1 (1%) | 0 (0%) | 0% |
| *Gemella* spp. | 0 (0%) | 0 (0%) | - |
| Group B microorganisms |  |  |  |
| *S. epidermidis* | 18 (23%) | 19 (61%) | 51% |
| Coagulase negative staphylococci other than *S. lugdunensis* and *S. epidermidis* | 6 (8%) | 1 ()3% | 14% |
| *C. acnes* | 1 (1%) | 2 (3%) | 67% |
| *P. aeruginosa* | 23 (30%) | 2 ()7% | 8% |
| *S. marcescens* | 16 (21%) | 0 (0%) | 0% |
| Non-tuberculous mycobacteria | 0 (0%) | 0 (0%) | - |
| *Candida* spp. | 20 (26%) | 7 (23%) | 26% |

Data are depicted as number (percentage)

**Supplementary Table 3.** Diagnostic performance of the three versions of the Duke clinical criteria for the diagnosis of IE by measuring the level of agreement between the reference standard and definite/possible IE cases

|  | **Sensitivity**  **% (95% CI)** | **Specificity**  **% (95% CI)** | **PPV**  **% (95% CI)** | **NPV**  **% (95% CI)** | **Accuracy**  **% (95% CI)** |
| --- | --- | --- | --- | --- | --- |
| 2015 Duke-ESC | 67 (54-78) | 94 (92-96 | 65 (54-74) | 95 (93-96) | 91 (87-93) |
| 2023 Duke-ISCVID | 92 (82-97) | 69 (64-74) | 32 (29-36) | 98 (96-99) | 72 (68-76) |
| 2023 Duke-ESC | 79 (67-89) | 92 (89-94) | 61 (52-69) | 97 (95-98) | 90 (87-93) |

ESC: European Society of Cardiology; IE: infective endocarditis; ISCVID: International Society of Cardiovascular Infectious Disease
